# Redox-Mediated Mitochondrial Dysfunction as a Common Pathogenic Axis in Acute Kidney Injury and Chronic Kidney Disease

**DOI:** 10.3390/biom16081148

**Published:** 2026-08-07

**Authors:** Ewelina Młynarska, Kinga Bojdo, Katarzyna Hossa, Wiktoria Lisińska, Katarzyna Krawiranda, Natalia Krupińska, Natalia Kustosik, Anna Wieczorek, Jacek Rysz, Beata Franczyk

**Affiliations:** 1 Department of Nephrocardiology, Medical University of Lodz, 90-419 Lodz, Poland; 2 Department of Nephrology, Hypertension and Internal Medicine, Medical University of Lodz, 90-549 Lodz, Poland

**Keywords:** acute kidney injury, chronic kidney disease, diabetic kidney disease, reactive oxygen species, reductive stress, biomarkers, mitochondrial bioenergetics, mitophagy, targeted therapies

## Abstract

Acute kidney injury (AKI), chronic kidney disease (CKD), and diabetic kidney disease (DKD) are interconnected disorders linked by shared mechanisms involving redox imbalance and mitochondrial dysfunction. This review summarizes current evidence on the role of excessive reactive oxygen species (ROS) production, impaired oxidized nicotinamide adenine dinucleotide (NAD^+^) metabolism, altered mitochondrial bioenergetics, disrupted mitochondrial dynamics, and defective mitochondrial quality control pathways, including mitophagy and the mitochondrial unfolded protein response (UPR^mt^), in kidney disease progression. Experimental and clinical studies indicate that these mechanisms contribute to inflammation, fibrosis, apoptosis, and maladaptive repair, promoting progression from AKI to CKD and worsening DKD. The review also discusses emerging biomarkers and therapeutic strategies targeting oxidative stress and mitochondrial dysfunction. Overall, redox-mediated mitochondrial injury represents a shared pathogenic axis and a potential therapeutic target across kidney diseases.

## 1. Introduction

Over the past few decades, significant progress has been made in understanding AKI and in standardizing its definition. Previously, the use of multiple, inconsistent criteria limited comparisons between studies. This led to the development of consensus classifications, culminating in the 2012 Kidney Disease: Improving Global Outcomes (KDIGO) definition. According to KDIGO, AKI is diagnosed based on a rapid increase in serum creatinine (≥0.3 mg/dL within 48 h or ≥1.5 times baseline within 7 days) and/or reduced urine output (<0.5 mL/kg/h for at least 6 h) [1].

AKI is now recognized as a common and serious condition, affecting up to 20% of hospitalized patients and about half of those in intensive care units. It occurs predominantly in older individuals with comorbidities, often in the setting of pre-existing CKD. Despite advances in care, severe AKI remains associated with high acute mortality. Furthermore, epidemiological data highlights that survivors carry a heavily elevated long-term risk of developing progressive CKD and subsequent cardiovascular complications. Given this clinical significance, AKI is no longer viewed as an isolated event, but rather as a critical initiating component in the continuum of chronic kidney disorders [1]. Beyond its diagnostic role, the KDIGO classification has become the most widely used framework in clinical practice and research, facilitating comparisons between studies and enabling standardized assessment of AKI severity. Importantly, increasing AKI stage is consistently associated with worse clinical outcomes, including higher in-hospital mortality, prolonged hospitalization, greater healthcare resource utilization, and an increased likelihood of readmission, underscoring the prognostic value of AKI staging [2].

In parallel with advances in the understanding of AKI, CKD has also been more precisely defined and systematically classified. The updated 2024 KDIGO guideline provides an evidence-based framework for the evaluation and management of CKD, incorporating significant progress achieved over the past decade in lifestyle interventions, pharmacotherapy, and diagnostic approaches. These developments have expanded opportunities for more effective treatment and monitoring, while emphasizing the need to integrate novel therapies with established management strategies in a patient-centered manner. CKD is defined as abnormalities in kidney structure or function persisting for at least 3 months, with implications for health. Its classification is based on three key components: cause, level of kidney function assessed by glomerular filtration rate, and degree of albuminuria, collectively referred to as the CGA system. This framework, introduced in earlier KDIGO guidelines and retained in updated recommendations, provides the basis for risk stratification, clinical decision-making, and research. Importantly, both reduced GFR and increased albuminuria independently predict adverse outcomes, including disease progression, cardiovascular events, mortality, kidney failure, and AKI. CKD represents a major and growing global health burden, affecting hundreds of millions of individuals worldwide. Its prevalence is driven in large part by common conditions such as hypertension and diabetes, and its impact is particularly pronounced in regions with limited healthcare resources. Despite increased awareness, early detection remains suboptimal, and many individuals are diagnosed at advanced stages. Screening efforts are therefore primarily focused on high-risk populations and rely on the assessment of GFR and albuminuria. Given its high prevalence, complex clinical course, and strong association with cardiovascular disease and kidney failure, CKD constitutes a central component of the spectrum of kidney disorders [3].

Building on the strong relationship between CKD and metabolic disorders, DKD represents one of the most common and clinically significant forms of chronic kidney injury. It develops in a substantial proportion of individuals with diabetes, with more than one-quarter of patients affected and up to 40% developing CKD over their lifetime. As the global prevalence of diabetes continues to rise, the burden of DKD has increased accordingly, making it the leading cause of kidney failure requiring dialysis or transplantation worldwide. DKD is typically defined by the presence of persistent reductions in estimated eGFR (eGFR < 60 mL/min/1.73 m^2^), albuminuria (albumin-to-creatinine ratio ≥ 30 mg/g), or other markers of kidney damage, present for at least 3 months. Importantly, the condition is often asymptomatic in its early stages and is most often detected through routine screening, particularly in high-risk populations such as individuals with type 1 or type 2 diabetes. Both the American Diabetes Association and KDIGO recommend regular assessment of kidney function and albuminuria to enable early diagnosis and risk stratification. Given its high prevalence, asymptomatic nature in early stages, and strong association with both CKD progression and cardiovascular complications, DKD represents a critical link within the continuum of kidney diseases. Together with AKI and CKD, it highlights the importance of shared pathogenic mechanisms, including redox-mediated mitochondrial dysfunction, which may underlie the development and progression of kidney injury across these interconnected conditions [4]. Recent ADA and KDIGO recommendations emphasize that the management of DKD should extend beyond glycemic control and adopt a comprehensive, patient-centered approach aimed at preserving both kidney and cardiovascular function. Current evidence supports the combined use of lifestyle interventions together with therapies targeting the renin–angiotensin system, sodium–glucose cotransporter-2 (SGLT2) inhibitors, glucagon-like peptide-1 (GLP-1) receptor agonists, and, in selected patients, nonsteroidal mineralocorticoid receptor antagonists. This integrated strategy reflects the growing recognition that early, multifactorial intervention is essential to slowing disease progression and reducing cardiovascular risk [3,4].

Mitochondria are essential regulators of kidney function, as they provide the energy required for highly demanding processes such as solute reabsorption, maintenance of ion gradients, and cellular homeostasis. Beyond their bioenergetic role, mitochondria are also central hubs of metabolic activity, coordinating pathways such as fatty acid β-oxidation, the tricarboxylic acid (TCA) cycle, and oxidative phosphorylation (OXPHOS). During these processes, ROS are continuously generated. Under physiological conditions, ROS act as important signaling molecules, modulating cellular pathways through redox-sensitive post-translational modifications (PTMs) of proteins and thereby regulating cell survival, proliferation, and metabolism [5].

However, in kidney diseases, this tightly controlled balance is disrupted. Excessive ROS production leads to oxidative stress, which damages mitochondrial components, including lipids, proteins, and mitochondrial DNA, and alters key metabolic pathways. As a result, mitochondrial bioenergetics become impaired, with reduced efficiency of OXPHOS, disturbances in the TCA cycle, and defective fatty acid β-oxidation. These alterations are accompanied by changes in mitochondrial dynamics, including increased fission, reduced fusion, impaired biogenesis, and dysregulated mitophagy, ultimately promoting cell injury and death. Importantly, redox-sensitive signaling pathways play a critical role in mediating these processes. ROS-induced modifications of specific amino acid residues, particularly cysteine and methionine, can reversibly or irreversibly alter protein function, thereby influencing key cellular pathways involved in inflammation, fibrosis, and apoptosis. While reversible oxidative modifications contribute to physiological signaling, persistent oxidative conditions lead to irreversible damage and loss of cellular function. In addition, crosstalk between mitochondrial ROS and other sources, such as NOXs, further amplifies oxidative stress, creating a self-perpetuating cycle of mitochondrial dysfunction. These mechanisms are increasingly recognized as central to the pathogenesis of both acute and chronic kidney diseases. Mitochondrial dysfunction driven by redox imbalance not only contributes to the development of AKI but also plays a pivotal role in its progression to CKD. Similar alterations are observed in diabetic kidney disease, where metabolic and oxidative stress further exacerbate mitochondrial impairment. Thus, redox-mediated mitochondrial dysfunction represents a unifying pathogenic axis linking AKI, CKD, and diabetic kidney disease, highlighting its importance as a potential target for therapeutic intervention [5,6]. Growing evidence indicates that persistent mitochondrial dysfunction is not merely a consequence of kidney injury but also a key driver of maladaptive repair and disease progression. Disruption of mitochondrial homeostasis, including impaired bioenergetics, altered mitochondrial quality control, and sustained oxidative stress, contributes to tubular injury, inflammation, and fibrosis, thereby linking acute and chronic forms of kidney disease. These findings have identified mitochondria as promising therapeutic targets for limiting renal damage and improving long-term kidney outcomes [7].

This review outlines the role of redox imbalance in kidney diseases, followed by its impact on mitochondrial function and quality control mechanisms. It then highlights shared pathogenic pathways linking AKI, CKD, and diabetic kidney disease, and concludes with potential therapeutic strategies targeting redox and mitochondrial dysfunction.

## 2. Search Strategy and Selection Criteria

Data for this review were identified through a comprehensive search of electronic databases, including PubMed, Scopus, and Google Scholar, using a combination of keywords such as acute kidney injury, chronic kidney disease, diabetic kidney disease, reactive oxygen species, reductive stress, biomarkers, mitochondrial bioenergetics, mitophagy, and targeted therapies to capture literature published between 2001 and 2026. While the chronological range encompasses over two decades, the selection process was strictly weighted toward the most recent advancements from 2020 to 2026 to ensure the review reflects the current understanding of molecular and metabolic pathways and the latest cellular protective interventions. Inclusion criteria prioritized high-impact clinical practice guidelines, expert consensus statements, and fundamental mechanistic studies describing the transition from acute tissue injury to chronic fibrotic pathways. A limited number of seminal historical publications were retained only where they provide the indispensable foundation for understanding early pathological mechanics and baseline cellular signaling, which remain essential to current research. Exclusion criteria were rigorously applied to ensure data integrity; specifically, studies were excluded if they featured insufficient detail regarding molecular interventions, lacked clear experimental validation, or were not published in English. Additionally, reports focusing on redundant molecular targets without novel mechanistic insights were omitted. The final evidence base was synthesized into a cutting-edge overview that focuses on the predictive power of emerging biomarkers and the critical steps required for their successful clinical translation into precision renal medicine.

## 3. Redox Imbalance in Kidney Disease

### 3.1. Reduced Nicotinamide Adenine Dinucleotide (NADH) Reductive Stress

Redox imbalance in disease states is closely linked to alterations in NAD^+^ metabolism, as NAD^+^ acts as a key regulator of cellular physiological processes. As an essential redox cofactor, NAD^+^ accepts electrons during metabolic pathways such as glycolysis, the TCA cycle, and fatty acid oxidation, leading to the formation of NADH, which is subsequently reoxidized to sustain cellular redox homeostasis. Under physiological conditions, the NAD^+^/NADH ratio is tightly regulated through coordinated processes of biosynthesis, recycling, and NADH oxidation [8].

However, under stress conditions, increased activity of NAD^+^-consuming enzymes, such as poly(ADP-ribose) polymerases (PARPs) and CD38, leads to NAD^+^ depletion, thereby disrupting the NAD^+^/NADH ratio and shifting cellular redox balance toward a more reduced cellular state [9]. Importantly, reduced NAD^+^ availability limits the activity of NAD^+^-dependent enzymes, including sirtuins, which are involved in the regulation of cellular metabolism and stress responses [8].

Such disturbances are increasingly recognized as early events in the pathogenesis of major kidney diseases. In AKI, a rapid decline in NAD^+^ levels, driven by both impaired biosynthesis and increased consumption, is associated with alterations in cellular redox homeostasis, including a decreased NAD^+^/NADH ratio. In CKD, sustained metabolic stress, inflammation, and altered substrate utilization contribute to persistent disruption of redox balance. Renal tubular epithelial cells, which rely heavily on oxidative metabolism to sustain their high energetic demand, are particularly susceptible to such disturbances [10,11]. In DKD, hyperglycemia-driven metabolic changes further enhance NADH production, reinforcing the shift toward a reduced intracellular environment [12]. Such disturbances may also contribute to persistent metabolic alterations and long-term cellular reprogramming [13].

At the mitochondrial level, reductive stress is characterized by an excess of reducing equivalents, particularly NADH and Nicotinamide Adenine Dinucleotide Phosphate (NADPH), which disrupts redox homeostasis and impairs efficient electron transfer within the electron transport chain (ETC). In addition, NADPH plays a central role in maintaining intracellular antioxidant defense by supporting glutathione- and thioredoxin-dependent redox systems [14]. Mitochondrial nicotinamide nucleotide transhydrogenase (NNT) contributes to the maintenance of the mitochondrial NADPH pool, thereby supporting antioxidant capacity under conditions of oxidative stress [14]. Excess NADH may promote electron accumulation at complexes I and III, favoring electron leakage and subsequent ROS generation. Consequently, reductive stress has been proposed to contribute to oxidative injury and mitochondrial dysfunction; however, direct causal evidence supporting this mechanism in human kidney disease remains largely inferential [14].

### 3.2. Transition to Oxidative Stress

Persistent disturbances in cellular redox homeostasis may promote a transition from reductive stress toward oxidative stress, although this process is likely to be context-dependent and influenced by disease stage and metabolic conditions. This transition is characterized by excessive ROS accumulation and impaired antioxidant defense mechanisms. Although physiological levels of ROS are essential for intracellular signaling and cellular adaptation, prolonged metabolic stress and mitochondrial dysfunction may promote pathological ROS overproduction, resulting in oxidative damage to lipids, proteins, and DNA and ultimately contributing to progressive cellular injury in kidney diseases [15].

Excessive ROS production may arise from multiple intracellular sources, including mitochondria, NADPH oxidases (NOXs), xanthine oxidase, uncoupled endothelial nitric oxide synthase, and activated inflammatory cells. Among NOX isoforms, NOX4 is particularly abundant in renal tissue and contributes to oxidative injury through enhanced superoxide generation, mitochondrial dysfunction, and activation of inflammatory and profibrotic pathways [16]. Increased ROS generation and mitochondrial dysfunction may mutually reinforce each other, thereby sustaining oxidative stress and contributing to progressive cellular and tissue injury [17].

Under physiological conditions, cellular redox balance is preserved by antioxidant defense systems composed of both enzymatic and non-enzymatic components. Superoxide dismutase, catalase, peroxiredoxins, and glutathione peroxidases constitute the major antioxidant enzymes, whereas glutathione and thioredoxin play important roles in intracellular antioxidant protection and redox regulation [18]. In addition, nuclear factor erythroid 2-related factor 2 (Nrf2) plays a central role in the cellular antioxidant response by regulating the expression of multiple antioxidants and cytoprotective genes in response to oxidative stress. However, in AKI, CKD, and DKD, persistent ROS accumulation may overwhelm these protective mechanisms, leading to impaired antioxidant capacity and increased susceptibility to oxidative injury [19].

Excessive ROS production contributes to oxidative damage affecting membrane lipids, proteins, and DNA, thereby disrupting cellular and mitochondrial homeostasis. Persistent oxidative stress may impair mitochondrial function and bioenergetics, further promoting inflammation, fibrosis, and progressive kidney injury. In addition, mitochondrial damage may lead to the release of mitochondrial DNA (mtDNA), which may act as a damage-associated molecular pattern (DAMP), contributing to inflammatory signaling, immune cell recruitment, and progression of kidney injury [20]. Collectively, sustained oxidative stress and impaired antioxidant defenses contribute to mitochondrial dysfunction, inflammation, fibrosis, and progression of kidney disease [20].

### 3.3. Redox Signaling vs. Redox Damage

ROS are not exclusively detrimental. At physiological levels, they act as important mediators of redox signaling involved in cellular adaptation, proliferation, metabolism, and stress responses. Controlled ROS production regulates multiple signaling pathways through reversible oxidative modification of proteins and activation of redox-sensitive transcription factors, thereby contributing to the maintenance of cellular homeostasis [21].

In renal cells, low and tightly regulated ROS concentrations support adaptive signaling mechanisms required for metabolic regulation and stress responses. These effects are largely mediated by reversible oxidation of cysteine residues, which transiently modifies protein activity and intracellular signaling interactions [18]. However, the biological effects of ROS are highly dependent on their concentration, duration of exposure, and subcellular localization. Excessive or persistent ROS accumulation disrupts signaling specificity, promotes irreversible oxidative modifications of cellular macromolecules, and shifts redox regulation from adaptive signaling toward pathological oxidative damage [18].

**Figure 1:**
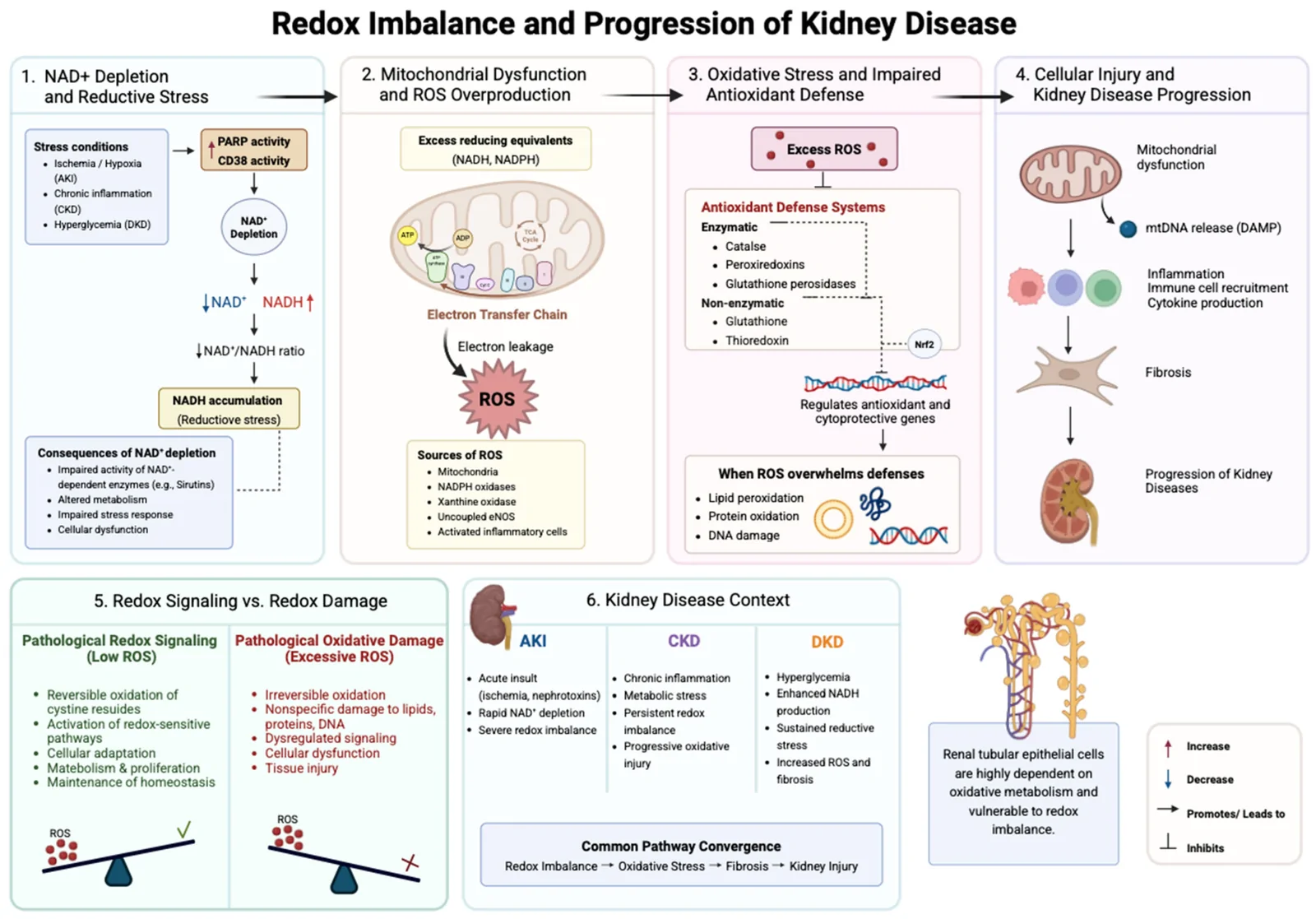
Redox Imbalance and Progression of Kidney Diseases. Pathological stress conditions such as ischemia, chronic inflammation, and hyperglycemia promote sustained ROS accumulation and cellular damage. This shared cascade drives mitochondrial dysfunction, inflammation, and fibrosis, serving as a converging pathogenic axis across acute, chronic, and diabetic kidney diseases. Symbols: →, promotes/leads to; ⊥, inhibits; ↑, increase; ↓, decrease; dashed lines indicate indirect regulatory interactions.Adapted from references [8,9,10,11,12,13,14,15,16,17,18,19,20,21,22]. Abbreviations: NAD^+^, oxidized nicotinamide adenine dinucleotide; NADH, reduced nicotinamide adenine dinucleotide; NADPH, reduced nicotinamide adenine dinucleotide phosphate; PARP, poly(ADP-ribose) polymerase; ROS, reactive oxygen species; mtDNA, mitochondrial DNA; DAMP, damage-associated molecular pattern; AKI, acute kidney injury; CKD, chronic kidney disease; DKD, diabetic kidney disease.

Importantly, physiological redox signaling depends on controlled ROS production and precise regulation of cellular responses [22]. Under pathological conditions, excessive or sustained ROS production disrupts redox regulation and promotes nonspecific oxidative damage to cellular components. As oxidative stress persists, cellular signaling pathways may become dysregulated and contribute to impaired cellular function. Thus, the transition from physiological redox signaling to oxidative damage reflects the loss of redox homeostasis required for normal cellular processes [21]. Collectively, these mechanisms illustrate how disturbances in redox homeostasis contribute to mitochondrial dysfunction, oxidative injury, inflammation, and progression of kidney disease (Figure 1).

## 4. Mitochondrial Dysfunction as a Central Mechanism

### 4.1. Mitochondrial Bioenergetics in Kidney Cells

Kidney cells, particularly proximal tubular epithelial cells, exhibit one of the highest metabolic demands in the human body due to the continuous requirement for active solute transport and maintenance of electrolyte gradients. To sustain these energy-intensive processes, renal tubular cells rely predominantly on mitochondrial oxidative metabolism rather than glycolysis. Under physiological conditions, adenosine triphosphate (ATP) production is primarily driven by OXPHOS, in which electrons derived from NADH and flavin adenine dinucleotide (FADH_2_) are transferred through the ETC located within the inner mitochondrial membrane [23].

The ETC consists of complexes I–IV and ATP synthase (complex V), which together generate a proton gradient required for ATP synthesis. Complex I (NADH oxidoreductase) serves as a major entry point for electrons generated during the TCA cycle and fatty acid β-oxidation. During normal respiration, a small fraction of electrons escapes from complexes I and III, leading to physiological production of mitochondrial reactive oxygen species (mtROS), which function as signaling molecules involved in cellular adaptation and redox regulation [24]. However, under pathological conditions, impaired electron flow and excessive electron accumulation promote enhanced ROS generation, oxidative damage, and reduced ATP production.

Mitochondrial bioenergetic dysfunction is increasingly recognized as a hallmark of both acute and chronic kidney diseases. In AKI, ischemia, nephrotoxins, and sepsis rapidly impair mitochondrial respiration by disrupting ETC activity, reducing ATP availability, and promoting mitochondrial membrane depolarization [25]. Similarly, in CKD and DKD, chronic metabolic stress, inflammation, and sustained oxidative injury progressively alter mitochondrial substrate utilization and respiratory efficiency. Defective fatty acid β-oxidation, accumulation of metabolic intermediates, and impaired TCA cycle activity contribute to persistent energetic deficiency and tubular dysfunction [26].

Importantly, mitochondrial metabolism is not uniform across the nephron. Proximal tubular epithelial cells possess the highest mitochondrial density and rely predominantly on fatty acid β-oxidation coupled to oxidative phosphorylation, making them particularly susceptible to ischemic and metabolic injury. In contrast, distal tubular cells exhibit greater metabolic flexibility, whereas glomerular cells, including podocytes, mesangial cells, and endothelial cells, display distinct metabolic profiles that reflect their specialized physiological functions. Consequently, mitochondrial dysfunction affects different renal cell populations through cell type-specific mechanisms, although impaired bioenergetics, oxidative stress, and defective mitochondrial quality control remain common pathogenic features across kidney diseases [27,28,29].

Under stress conditions, different renal cell populations undergo distinct forms of metabolic reprogramming aimed at maintaining survival, with proximal tubular cells typically exhibiting the most pronounced shift from fatty acid oxidation toward glycolytic metabolism.

This process is characterized by reduced mitochondrial oxidative metabolism and increased dependence on glycolysis, even in oxygen-replete conditions. Although such a shift may initially serve as an adaptive response limiting ROS production, prolonged metabolic reprogramming contributes to maladaptive repair, fibrosis, and chronic kidney injury [30]. In particular, impaired fatty acid oxidation has emerged as a central mechanism promoting tubular lipid accumulation, inflammation, and fibrogenesis in CKD and DKD [25].

Moreover, alterations in mitochondrial metabolism influence multiple signaling pathways involved in inflammation, apoptosis, and redox regulation. Reduced NAD^+^ availability, excessive NADH accumulation, and impaired ETC activity collectively reinforce mitochondrial dysfunction and establish a self-perpetuating cycle of oxidative stress and bioenergetic failure. Consequently, this progressive metabolic insufficiency serves as a shared pathogenic mechanism underlying cellular injury across AKI, CKD, and DKD, although the degree and nature of mitochondrial vulnerability vary considerably among proximal tubular cells, distal tubular cells, podocytes, mesangial cells, and glomerular endothelial cells.

### 4.2. Mitochondrial Adaptation and Maladaptation

One of the key protective pathways is the UPR^mt^, which is activated in response to the accumulation of unfolded or damaged proteins within mitochondria. UPR^mt^ promotes the transcription of mitochondrial chaperones, proteases, and antioxidant enzymes aimed at restoring mitochondrial proteostasis and respiratory function [31]. Experimental studies suggest that transient activation of UPR^mt^ may attenuate oxidative injury and improve mitochondrial resilience in AKI models [32]. However, chronic activation may become insufficient to counteract ongoing mitochondrial damage during CKD progression.

Mitochondrial biogenesis represents another critical adaptive mechanism. This process is largely regulated by peroxisome proliferator-activated receptor gamma coactivator-1 alpha (PGC-1α), a master regulator coordinating mitochondrial replication, oxidative metabolism, and antioxidant defense [33]. In healthy kidneys, PGC-1α supports mitochondrial homeostasis and energy production, particularly in proximal tubular cells. During AKI, suppression of PGC-1α expression contributes to ATP depletion and impaired recovery, whereas restoration of mitochondrial biogenesis has been associated with improved renal repair [34]. Similar reductions in PGC-1α signaling have been observed in CKD and DKD, where sustained metabolic stress and inflammation impair mitochondrial renewal and promote fibrosis [35].

Importantly, adaptive mitochondrial responses may transition into maladaptive signaling when cellular stress becomes persistent. During severe or recurrent AKI, unresolved mitochondrial dysfunction prevents renal tubules from recovering their baseline energy capacity. This chronic energetic deficit forces cells into a persistent state of cellular senescence and apoptosis, establishing a metabolic environment that accelerates the progression toward chronic tissue remodeling [36].

### 4.3. Mitochondrial Dynamics: Fission, Fusion, and Network Integrity

Mitochondria form highly dynamic intracellular networks that continuously undergo fission and fusion processes to maintain structural integrity, bioenergetic efficiency, and cellular homeostasis. These mitochondrial dynamics are particularly important in kidney tubular epithelial cells, which depend heavily on efficient mitochondrial function to sustain high ATP demand. However, the consequences of altered mitochondrial dynamics differ among renal cell types; for example, excessive mitochondrial fragmentation contributes to apoptosis in proximal tubular cells, whereas in podocytes it disrupts cytoskeletal maintenance and filtration barrier integrity, and in glomerular endothelial cells it promotes oxidative stress and microvascular dysfunction.

Balanced regulation of mitochondrial fission and fusion enables adaptation to metabolic stress, facilitates removal of damaged mitochondria, and preserves mtDNA integrity [37].

Mitochondrial fusion is primarily mediated by mitofusin 1 and 2 (MFN1 and MFN2) located on the outer mitochondrial membrane, and optic atrophy protein 1 (OPA1) located on the inner mitochondrial membrane. Fusion promotes mixing of mitochondrial contents, including mtDNA and metabolic intermediates, thereby supporting respiratory efficiency and resistance to stress [38]. In contrast, mitochondrial fission is largely controlled by dynamin-related protein 1 (DRP1), which translocates from the cytosol to mitochondria to mediate mitochondrial fragmentation [39].

Under physiological conditions, coordinated fission and fusion maintain mitochondrial quality control and facilitate adaptation to fluctuating metabolic demands. However, kidney injury is frequently associated with excessive mitochondrial fission and fragmentation. In AKI, IRI and nephrotoxic stress activate DRP1-dependent mitochondrial fragmentation, leading to mitochondrial depolarization, increased ROS production, cytochrome c release, and apoptosis of tubular epithelial cells [40]. Experimental inhibition of DRP1 has been shown to attenuate mitochondrial injury and improve renal function in several AKI models [39].

Altered mitochondrial dynamics also contribute to chronic kidney diseases. In CKD and DKD, persistent oxidative stress and metabolic dysfunction impair mitochondrial fusion and promote sustained fragmentation. Reduced expression of MFN2 and OPA1 has been associated with defective mitochondrial networking, impaired mitophagy, and progressive tubular injury [30]. Moreover, disrupted mitochondrial dynamics contribute to inflammation, fibrosis, and cellular senescence, thereby reinforcing disease progression. The major mitochondrial alterations contributing to bioenergetic dysfunction, oxidative stress, and progression of kidney disease are summarized in Table 1.

## 5. Mitochondrial Quality Control

### 5.1. Mitophagy in Kidney Disease

#### 5.1.1. Physiological Role and Regulation

Mitophagy is a selective form of autophagy responsible for the removal of damaged or dysfunctional mitochondria, thereby maintaining mitochondrial homeostasis and cellular survival. This process is essential for mitochondrial quality control, especially in highly energy-dependent kidney tubular cells. Mitophagy is mainly regulated by the PTEN-induced kinase 1 (PINK1)–parkin pathway and receptor-mediated mechanisms involving proteins such as BCL2 interacting protein 3 (BNIP3), BCL2 interacting protein 3 Like (BNIP3L), FUN14 domain containing 1 (FUNDC1) and SMAD specific E3 ubiquitin protein ligase 1 (SMURF1). Following mitochondrial damage or depolarization, PINK1 accumulates on the outer mitochondrial membrane and recruits parkin, which promotes ubiquitination of mitochondrial proteins and their recognition by autophagosomes. Mitophagy receptors can also directly interact with microtubule-associated protein 1 light chain 3 B (LC3B) to facilitate autophagosome formation and lysosomal degradation of damaged mitochondria [34].

Mitochondrial ROS production is closely linked to the regulation of mitophagy and mitochondrial quality control in kidney cells. During oxidative phosphorylation, electron leakage from complexes I and III of the ETC generates superoxide anions, which represent a major source of mitochondrial ROS. Under physiological conditions, moderate ROS levels participate in intracellular signaling, whereas excessive ROS production induces mitochondrial dysfunction, oxidative damage to proteins, lipids and mtDNA, and loss of mitochondrial membrane potential. To counteract oxidative stress, mitochondria rely on enzymatic and non-enzymatic antioxidant defense systems, including superoxide dismutase, catalase, glutathione and thioredoxin-dependent pathways. Mitophagy serves as an adaptive mechanism limiting oxidative injury through selective elimination of damaged mitochondria and maintenance of mitochondrial integrity. ROS-induced mitochondrial depolarization promotes activation of mitophagy pathways and recruitment of autophagic machinery [39]. In addition to the PINK1-parkin pathway, mitophagy can also be regulated through parkin RBR E3 ubiquitin protein ligase (PRKN)-independent mechanisms involving BNIP3, FUNDC1, autophagy stabilizing regulator of BECN1 (AMBRA1) and cardiolipin-mediated signaling. Cardiolipin translocation from the inner to the outer mitochondrial membrane facilitates interaction with LC3 proteins and promotes autophagosome formation around injured mitochondria. Furthermore, several ubiquitin ligases, including mitochondrial E3 ubiquitin protein ligase 1 (MUL1) and HECT, UBA, and WWE domain containing E3 ubiquitin protein ligase 1 (HUWE1), contribute to mitochondrial ubiquitination and regulation of mitophagy independently of parkin. The balance between ROS generation and mitophagy activation appears to be essential for renal cell survival. While physiological mitophagy protects kidney cells from oxidative stress and mitochondrial damage, excessive or prolonged activation may contribute to mitochondrial loss, ATP depletion and tissue injury. These findings indicate that precise regulation of mitophagy and redox homeostasis is critical for maintaining mitochondrial function in kidney disease [39].

#### 5.1.2. Impaired Mitophagy and Disease Progression

Disturbances in mitophagy have been strongly associated with the development and progression of both AKI and CKD. Experimental studies demonstrated increased mitophagy in kidney tubular cells during the early stages of ischemic, septic and nephrotoxic AKI, suggesting a protective response against mitochondrial damage, ROS accumulation and apoptosis. Deficiency of mitophagy-related proteins such as PINK1, parkin or BNIP3 aggravated mitochondrial dysfunction, inflammation and tubular injury. However, prolonged or severe injury may impair mitophagy, leading to accumulation of damaged mitochondria and worsening kidney damage. Impaired mitophagy has also been observed in diabetic and non-diabetic CKD. Reduced expression of PINK1, parkin and optineurin in kidney tubular cells and podocytes was associated with mitochondrial dysfunction, oxidative stress, inflammasome activation and fibrosis. Experimental restoration of mitophagy attenuated cellular injury and improved mitochondrial function. These findings indicate that mitophagy plays a protective, cell-type-specific role in kidney disease and may represent a potential therapeutic target for preventing kidney injury and promoting repair [34].

Additional evidence highlights the context-dependent role of mitophagy in specific forms of AKI. In IRI, activation of BNIP3-, FUNDC1- and PINK1-parkin-mediated pathways was associated with improved mitochondrial quality control, reduced oxidative damage and enhanced tubular cell survival. Experimental inhibition of these pathways resulted in accumulation of fragmented mitochondria, increased inflammation and aggravated renal dysfunction. Moreover, mitophagy appears to interact closely with mitochondrial fission mechanisms, particularly through DNM1L/DRP1 signaling, suggesting coordinated regulation of mitochondrial turnover during renal stress. In septic AKI, mitophagy is activated at early stages of injury as an adaptive response to inflammation-induced mitochondrial damage and ATP depletion. Activation of the PINK1-parkin pathway promoted removal of dysfunctional mitochondria and contributed to attenuation of inflammatory responses in tubular epithelial cells. Similar protective effects were observed in cisplatin- and contrast-induced AKI, where mitophagy reduced mitochondrial ROS generation, apoptosis and inflammasome activation. Pharmacological agents enhancing mitophagy, including paricalcitol and tetramethylpyrazine, demonstrated renoprotective effects in experimental models of contrast-induced nephropathy. Despite predominantly protective effects, excessive or dysregulated mitophagy may also contribute to kidney injury under certain conditions. Some studies suggested that overactivation of mitophagy could exacerbate tubular damage through enhanced mitochondrial loss and activation of pro-fibrotic signaling pathways. These observations indicate that balanced regulation of mitophagy is critical for maintaining mitochondrial integrity and renal cell survival during kidney injury and repair [41].

In diabetic kidney disease, impaired mitophagy contributes to mitochondrial dysfunction, oxidative stress and progression of renal injury. High-glucose conditions were associated with reduced expression of key mitophagy-related proteins, including PINK1, parkin, LC3II, Beclin1 and Atg5, in tubular cells, podocytes, mesangial cells and glomerular endothelial cells. Suppression of mitophagy promoted mitochondrial fragmentation, accumulation of damaged mitochondria, increased mtROS production and activation of inflammatory pathways such as the NLR Family Pyrin Domain Containing 3 (NLRP3) inflammasome. Experimental models also demonstrated that impaired mitophagy was associated with fibrosis, apoptosis, premature senescence of renal tubular epithelial cells and deterioration of renal function. Several regulatory molecules involved in DKD-associated mitophagy dysfunction have been identified. Reduced expression of optineurin, progranulin and FoxO1 was linked to impaired PINK1–parkin signaling and defective mitochondrial clearance, whereas restoration of these pathways improved mitochondrial function and reduced oxidative stress. Alterations in mitophagy-related signaling pathways, including PI3K/AKT/mTOR and Nrf2/ARE, were also implicated in diabetic renal injury. Interestingly, studies suggest that mitophagy may initially increase during early diabetes as an adaptive response to eliminate dysfunctional mitochondria, but becomes progressively impaired during disease progression, leading to accumulation of mitochondrial damage and cell death. Current evidence indicates that mitophagy regulation in DKD is highly cell-type-specific and incompletely understood. Advances in multiomics technologies, metabolic analyses and kidney organoid models may improve understanding of mitochondrial quality control mechanisms and facilitate development of targeted therapies aimed at restoring mitophagy and mitochondrial homeostasis in DKD [40].

### 5.2. Mitochondrial Unfolded Protein Response

The UPR^mt^ is triggered when the amount of unfolded or misfolded proteins exceeds the capacity of this quality control system [34]. It was first described in 2002 and showed that the buildup of unfolded proteins in the mitochondrial matrix caused a transcriptional response specific to mitochondria, marked by the upregulation of nuclear genes encoding mitochondrial chaperones and proteases, such as HSP60, HSP10, mtDnaJ, and ClpP [29]. This response is now recognized as a central component of mitochondrial quality control, acting to enhance the protein-folding capacity, remove damaged proteins, and sustain fitness under stress conditions [34,41]. As established in Section 3.1, this mitochondrial oxidative metabolism is essential for normal renal function due to the unique energetic constraints of proximal tubular epithelial cells [42].

UPR^mt^ can be triggered by several forms of mitochondrial proteotoxic stress, including accumulation of misfolded mitochondrial proteins, impaired mitochondrial protein import, respiratory chain dysfunction, excessive ROS production, mtDNA damage and broader mitochondrial dysfunction in mitochondrial redox and metabolic homeostasis [43]. In mammalian cells, UPR^mt^ is increasingly viewed not as a single linear pathway, but as a multilayered adaptive network composed of partially overlapping signaling modules. ATF5 is considered a central effector of the canonical transcriptional response: as mitochondrial stress impairs its import into mitochondria, thereby promoting its nuclear accumulation and the induction of genes involved in mitochondrial proteostasis, including chaperones and proteases [41,44]. In parallel, mitochondrial dysfunction can be relayed through the DELE1-HRI-eIF2α-ATF4/CHOP axis, which functionally connects UPR^mt^ with the integrated stress response and broadens the adaptive transcriptional program [44,45]. In addition, factors such as HSF1, the SIRT3/FOXO3a antioxidant axis, and proteostasis regulators including LONP1 further modulate the magnitude and outcome of the response by influencing protein quality control, antioxidant defense, and mitochondrial recovery [44,46,47].

The contribution of UPR^mt^ to kidney disease appears to be highly context-dependent, with evidence supporting both adaptive and maladaptive effects across AKI, CKD and DKD. In acute or transient stress settings, timely UPR^mt^ activation may help preserve mitochondrial integrity by enhancing protein folding and proteolytic capacity, maintaining membrane potential, and promoting tubular cell survival [44,48]. By contrast, persistent or excessive UPR^mt^ activation may reflect unresolved mitochondrial injury and ongoing proteotoxic stress. Under such conditions, prolonged stress signaling may become maladaptive, particularly when accompanied by sustained ROS accumulation, defective mitophagy, and persistent inflammatory or profibrotic responses, thereby contributing to tubular injury and fibrotic remodeling [46,48].

In AKI, mitochondrial dysfunction is an early and central event, particularly in ischemia–reperfusion and nephrotoxic injury, where ATP depletion, respiratory chain impairment, oxidative stress, and mitochondrial structural damage contribute to tubular epithelial cell injury [49]. Experimental studies in renal ischemia–reperfusion and cisplatin-induced AKI models further indicate that disturbed mitochondrial homeostasis directly promotes tubular damage and loss of renal function. Although direct AKI-specific evidence for UPR^mt^ remains limited, available data suggest that early activation of this pathway may be protective [50]. In a murine renal ischemia–reperfusion model, short-term time-restricted feeding reduced renal injury and was associated with transient UPR^mt^ induction, improved antioxidant defense, reduced mitochondrial fragmentation, and more favorable later remodeling [50,51]. These observations support the concept that UPR^mt^ serves as an early adaptive response to acute mitochondrial stress. However, when the insult is severe or sustained, this compensatory mechanism may be overwhelmed, promoting progression toward tubular cell death, maladaptive repair, and persistent renal injury [52].

The role of UPR^mt^ in CKD appears more complex than in AKI, as mitochondrial stress is chronic. Current evidence points to a predominantly protective role of the mitochondrial protease LONP1.

In both human and experimental CKD, LONP1 expression is reduced, and its loss worsens mitochondrial dysfunction, renal injury, and fibrosis, whereas tubular Lonp1 overexpression is protective [53]. One proposed mechanism is the accumulation of mitochondrial HMGCS2 in the setting of LONP1 deficiency, which further impairs mitochondrial homeostasis [53]. Consistent with this, pharmacological restoration of LONP1 ameliorates fibrosis in both CKD and aging-related renal injury models [54]. More recently, reduced endothelial LONP1 has also been shown to promote oxidative stress, inflammation, and glomerulosclerosis, extending the relevance of this pathway beyond tubular cells [55]. Overall, these findings support the idea that defective mitochondrial stress adaptation contributes to CKD progression.

In patients with DKD, increased expression of ATF5, HSP60, and LONP1 has been observed and shown to correlate with tubular injury, apoptosis, and tubulointerstitial fibrosis. These alterations were attenuated by ATF5 knockdown, suggesting that although UPR^mt^ activation may initially represent an adaptive response, its persistent activation under chronic diabetic stress may become maladaptive [48]. Mechanistically, chronic hyperglycemia may sustain mitochondrial metabolic overload and proteotoxic stress, leading to persistent activation of UPRmt. When this response falls to restore mitochondrial proteostasis, sustained UPRmt signaling may coexist with increased mitochondrial ROS production, defective mitochondrial quality control, tubular apoptosis, inflammatory signaling, and maladaptive repair, thereby contributing to extracellular matrix accumulation and tubulointestinal fibrosis [48,56]. This interpretation is further supported by more recent findings involving the mitochondrial protease ClpP. In DKD models, ClpP expression was increased in parallel with ATF5, HSP60, and HSP10 upregulation, whereas ClpP deficiency was associated with reduced mitochondrial ROS production, apoptosis, and tubulointerstitial injury [56]. Taken together, these findings suggest that in DKD, sustained activation of UPR^mt^-related pathways may shift from an initially adaptive response toward a maladaptive program associated with persistent mitochondrial stress, tubular injury, and progression of tubulointerstitial fibrosis. However, current mechanistic evidence in DKD is still relatively limited and comes primarily from experimental models.

To clarify context-dependent role of UPRmt, Table 2 summarizes how its function may shift from adaptive to maladaptive depending on the disease context and duration of mitochondrial stress.

### 5.3. Crosstalk Between Redox and Mitochondrial QC

Mitophagy and the UPR^mt^ are mechanistically distinct quality control pathways, but both can be disrupted by redox imbalance, which also drives the mitochondrial damage they are meant to resolve. Redox imbalance regulates mitochondrial quality control by activating both the UPR^mt^ and mitophagy. Studies showed that mitochondrial proteotoxic stress increases ROS, which induces UPR^mt^ markers and stimulates ROS-dependent mitophagy. SIRT3 acts as a central mediator of this response by activating FOXO3A-dependent antioxidant defense and supporting LC3B-mediated mitophagy. However, when SIRT3 is inhibited, mitophagy and antioxidant protection fail, mitochondrial ROS rises further, membrane potential collapses, and cell death increases. This suggests that redox stress can initially activate mitochondrial quality control, but if the SIRT3-dependent adaptive response is insufficient, it may contribute to a self-amplifying cycle of mitochondrial injury [47].

When quality control fails, damaged mitochondria accumulate and continue leaking electrons from the destabilized respiratory chain, generating further ROS and deepening the dysfunction that impaired clearance in the first place. Experimental evidence supports this directly, impaired mitophagy, whether through BNIP3 silencing or PINK1/Parkin deficiency, consistently results in mitochondrial ROS accumulation compounded by mtROS-driven NLRP3 inflammasome activation, which independently promotes tubular cell death and interstitial fibrosis, adding an inflammatory dimension to an already self-sustaining cycle of injury [57,58].

The pattern of this quality control failure differs by disease timeline. In AKI, the initial redox insult is acute and intense; if the injury severely overwhelms mitophagy and the UPR^mt^, the structural clearance machinery breaks down completely. This leaves fragmented, dysfunctional mitochondria to continuously leak electrons and release immunogenic mtDNA into the cytosol, transforming an acute metabolic insult into a chronic, localized inflammatory driver [59]. In chronic settings, factors such as uremic toxins, chronic hyperglycemia, and lipotoxicity gradually impair quality control capacity [60]. In DKD, this chronic hyperglycemic stress drives a distinct pattern of dysfunction. While the initial activation of the ATF5-HSP60/LONP1 axis serves a protective role, its sustained upregulation eventually transitions into a maladaptive phase [48]. Notably, ATF5 inhibition at early timepoints exacerbated mitochondrial ROS and apoptosis, confirming that the initial UPR^mt^ activation is protective and that the maladaptive shift is specific to the chronic phase of hyperglycemic stress [48]. These distinct cellular timelines illustrate how overlapping quality control failures govern both acute structural damage and chronic tissue remodeling throughout the progression of renal diseases.

## 6. The Shared Mitochondrial–Redox Axis in AKI and CKD

While AKI and CKD were long regarded as separate pathologies with the expectation of full recovery after acute episodes, current perspectives emphasize their integrated nature. A robust bidirectional relationship exists between these syndromes: [61] underlying CKD increases baseline susceptibility to acute episodes, while unresolved AKI serves as a primary driver of chronic, progressive nephron loss. Not all patients who experience AKI subsequently develop chronic sequelae, indicating a variable threshold for long-term tissue remodeling [62]. However, the global clinical impact of this continuum remains stark. A systematic review and meta-analysis conducted by Veltkamp et al. (2025), including almost 2 million individuals, demonstrated that patients with AKI had a significantly increased risk of developing CKD, with an incidence of 25.8% compared with 8.7% in individuals without AKI (HR 2.36; 95%CI 1.77–2.94), as well as a higher risk of CKD progression (43.1% vs. 35.6%; HR 1.83; 95%CI 1.26–2.40) [63]. Conversely, a meta-analysis showed that a lower baseline estimated glomerular filtration rate (eGFR) was strongly associated with an increased risk of subsequent AKI. Compared with an eGFR of 80 mL/min/1.73 m^2^, the adjusted hazard ratio for AKI at an eGFR of 45 mL/min/1.73 m^2^ was 3.35 (95%CI 2.75–4.07) [64]. On a cellular level, this continuum is sustained because the initial acute structural damage triggers persistent endothelial injury, capillary rarefaction, and the activation of robust pro-fibrotic signaling pathways that ultimately culminate in widespread tubulointerstitial fibrosis [61].

AKI may result from a variety of causes, including reduced renal perfusion, intrinsic renal parenchymal disorders such as acute tubular necrosis, and obstruction of the urinary tract [65]. In AKI renal tubular epithelial cells are the primary site of injury, as they are particularly vulnerable to metabolic stress, ischemia, and redox disturbances [6]. Although this classification supports differential diagnosis, clinical AKI is most often multifactorial, with substantial overlap in underlying pathophysiological mechanisms [66]. Concurrently, diabetes and hypertension represent the leading global causes of CKD, with diabetes being the predominant contributor [67]. Although triggered by markedly distinct clinical insults—such as sudden perfusion deficits, nephrotoxicity, or chronic metabolic disturbances—these conditions converge upon a common final pathway. This shared pathogenic axis is fundamentally driven by redox imbalance and mitochondrial impairment [68].

Ischemia–reperfusion injury (IRI) represents a clinically relevant example of how acute mitochondrial–redox disturbances may initiate long-term kidney remodeling. Although renal function may initially recover, severe IRI can induce persistent structural alterations that promote progression from AKI to CKD [69,70,71]. According to Basile et al. (2001), these changes include persistent impairment of urinary concentrating ability, reduced peritubular capillary density, and progressive tubulointerstitial fibrosis, thereby driving the transition from acute injury to chronic renal dysfunction [72].

Alongside ischemic events, drug-induced nephrotoxicity represents another major cause of AKI, accounting for approximately 19–26% of cases in hospitalized patients [73]. Despite diverse initiating mechanisms, many nephrotoxic substances directly impair renal metabolic pathways, promoting acute tubular injury and contributing to the AKI-to-CKD continuum [74,75].

The mitochondrial–redox disruption is evident in drug-induced nephrotoxicity. Based on a case-crossover study among 1284 newly diagnosed AKI patients, NSAID use was associated with a 3.55-fold increased risk of AKI (95%CI 2.70–4.65) [76]. Ultimately, the persistent mitochondrial dysfunction and oxidative stress triggered by agents such as NSAIDs and cisplatin drive chronic tissue remodeling, transitioning acute cellular damage into sustained inflammation and interstitial fibrosis [74,77]. Consequently, drug-induced nephrotoxicity is increasingly recognized not only as a trigger for AKI but also as a contributor to the AKI-to-CKD continuum [61]. Environmental exposure to toxic heavy metals further illustrates this shared pathogenic axis. Although these toxicants differ in their primary cellular targets, they commonly induce oxidative stress and mitochondrial dysfunction, leading to tubular injury [78,79,80]. According to a systematic review and meta-analysis including 107,539 participants, exposure to heavy metals was also associated with increased risk of CKD-related markers, with cadmium linked to higher proteinuria risk (OR 1.35; 95%CI 1.13–1.61), lead associated with both reduced eGFR (OR 1.12; 95%CI 1.03–1.22) and increased proteinuria (OR 1.25; 95%CI 1.04–1.49), and arsenic associated with decreased eGFR (OR 1.55; 95%CI 1.05–2.28) [79].

The kidney’s high metabolic vulnerability during these exposures compounds the systemic burden of CKD. The resulting systemic oxidative stress heavily contributes to secondary cardiovascular complications, a trajectory uniquely exacerbated by non-mechanistic clinical factors including dietary restrictions, diuretic use, protein-energy wasting, and impaired intestinal absorption [81].

Hypertension is a major contributor to CKD and end-stage renal disease, largely driven by chronic hemodynamic and oxidative stress [82]. Rather than acting merely as a generic source of reactive species, Angiotensin II directly amplifies tissue injury by stimulating NADPH oxidase activity, establishing a self-perpetuating cycle linking renin–angiotensin–aldosterone system (RAAS) activation, mitochondrial dysfunction, and progressive renal damage [82]. Segment-specific differences in renal metabolism, mitochondrial substrate utilization, and alterations in the TCA cycle further accelerate this pathology, underscoring the need for targeted, nephron-specific therapeutic approaches [83]. Therapeutic strategies targeting RAAS, sodium-glucose cotransporter 2 (SGLT2), and mineralocorticoid receptor signaling mitigate this chronic oxidative and metabolic burden, thereby slowing disease progression [82].

Among the various causes of CKD, DKD represents the clinical model where metabolic-driven mitochondrial stress is most extensively characterized [84]. In the diabetic kidney, excessive mtROS overproduction serves as a unifying driver of progressive renal damage, contributing directly to podocyte apoptosis and loss [38,84]. In vivo, two-photon imaging using a mitochondria-targeted redox-sensitive biosensor revealed significantly elevated mtROS production in the kidneys of diabetic (db/db) mice. Furthermore, enhancing mitochondrial Complex I activity in podocytes successfully attenuated high glucose-induced mtROS generation, supporting the critical role of mitochondrial oxidative stress in diabetic nephropathy [85].

Moreover, because mtDNA is located near the primary site of mtROS generation, it is particularly susceptible to oxidative damage, including strand breaks and base modifications. In DKD, persistent hyperglycemia disrupts cellular bioenergetics and promotes mtROS overproduction with consequent mtDNA injury, as demonstrated in glomeruli from DBA/2J mice and streptozotocin-induced rat models [86].

Importantly, hypertension and diabetes frequently coexist, creating a synergistic effect on redox-mediated mitochondrial dysfunction. In type 2 diabetes, hypertension frequently precedes the onset of kidney disease, affecting up to 58–70% of newly diagnosed patients without proteinuria. Although its incidence is not strictly related to diabetes duration, it is strongly associated with impaired kidney function and increased cardiovascular risk [87,88]. This combined pathology is heavily driven by Angiotensin II-mediated oxidative stress. ATreatment with Angiotensin II receptor blockers in DKD patients significantly reduced oxidative stress and inflammatory markers (including urinary 8-epi-prostaglandin F2α and 8-hydroxydeoxyguanosine), along with decreased albumin and type IV collagen excretion. Notably, these effects occurred independently of blood pressure reduction and were more pronounced in patients with higher baseline oxidative stress, highlighting the shared pathogenic axis linking RAAS activation, oxidative injury, and disease progression [88].

## 7. Translational and Therapeutic Implications

### 7.1. Biomarkers for Mitochondrial Redox Dysfunction

The growing recognition of redox-mediated mitochondrial dysfunction as a central mechanism linking AKI, CKD, and DKD has stimulated interest in biomarkers reflecting oxidative stress, mitochondrial injury, and impaired mitochondrial quality control. Such biomarkers may improve early diagnosis, risk stratification, prediction of disease progression, and monitoring of therapeutic responses. Candidate biomarkers can be broadly categorized into markers of oxidative damage, mitochondrial dysfunction, redox imbalance, and mitochondrial quality control pathways [89].

Among biomarkers of oxidative stress, lipid peroxidation products such as malondialdehyde (MDA), F2-isoprostanes, and 4-hydroxynonenal (4-HNE) are frequently elevated in kidney diseases and reflect ROS-mediated membrane damage. Oxidative DNA injury may be assessed by 8-hydroxy-2′-deoxyguanosine (8-OHdG), which has been associated with tubular injury, diabetic nephropathy progression, and cardiovascular risk in CKD. Protein oxidation markers, including advanced oxidation protein products (AOPPs) and nitrotyrosine, also correlate with oxidative stress severity and inflammation [90,91].

Mitochondrial-specific biomarkers have attracted increasing attention because they may more directly reflect mitochondrial dysfunction. Circulating and urinary mtDNA are released during mitochondrial injury and have been associated with AKI severity, inflammation, and progression toward CKD. Elevated urinary mtDNA levels have been observed in ischemic AKI, septic AKI, and DKD, suggesting their potential utility as noninvasive markers of mitochondrial damage. Additional candidate biomarkers include mitochondrial transcription factor A (TFAM), cytochrome c, ATP synthase subunits, and proteins involved in mitochondrial dynamics such as DRP1 and MFN2 [92].

Disturbances in NAD^+^ metabolism may also provide clinically relevant biomarkers of redox dysfunction. Reduced intracellular NAD^+^ levels, altered NAD^+^/NADH ratios, and increased activity of NAD^+^-consuming enzymes including CD38 and PARPs have been described in experimental and clinical kidney disease. Furthermore, reduced expression of sirtuins, particularly SIRT1 and SIRT3, has been linked to impaired mitochondrial function, inflammation, and fibrosis [93].

Biomarkers related to mitochondrial quality control pathways are also emerging. Altered expression of PINK1, parkin, BNIP3, LC3-II, and ATF5 has been observed in AKI, CKD, and DKD models, reflecting dysregulation of mitophagy and UPR^mt^ activation. Increased circulating or tissue levels of HSP60, ClpP, and LONP1 may indicate persistent mitochondrial proteotoxic stress and maladaptive UPR^mt^ signaling [94].

Clinically, these biomarkers may provide several advantages over traditional renal markers such as serum creatinine and albuminuria, which primarily reflect functional decline rather than underlying cellular injury. Biomarkers of mitochondrial dysfunction and oxidative stress may allow earlier detection of kidney injury before overt loss of renal function occurs. In AKI, mitochondrial injury markers may help identify patients at risk of progression toward persistent renal dysfunction or CKD. In CKD and DKD, they may improve risk stratification, identify individuals with active mitochondrial stress, and facilitate monitoring of responses to targeted therapies [95].

Despite their promise, several limitations currently restrict routine clinical implementation. Many biomarkers lack disease specificity because oxidative stress and mitochondrial dysfunction occur in multiple systemic disorders. Substantial variability exists between studies regarding assay methods, sample handling, and reference ranges. Furthermore, many candidate biomarkers have been validated primarily in experimental models or small observational cohorts, while large prospective clinical studies remain limited. The dynamic and context-dependent nature of redox signaling additionally complicates interpretation, as transient ROS elevations may reflect adaptive signaling rather than pathological injury. Consequently, future research should focus on standardization of measurement techniques, validation in large patient populations, and integration of multimarker panels combining functional, inflammatory, and mitochondrial biomarkers to improve diagnostic and prognostic performance [96].

### 7.2. Targeting Redox Balance

Given the central role of oxidative stress and redox imbalance in mitochondrial dysfunction and kidney injury, therapeutic strategies aimed at restoring redox homeostasis have attracted considerable interest. These approaches include antioxidants, NAD^+^-restoring therapies, and modulators of redox-sensitive signaling pathways [97].

Classical antioxidants such as vitamins C and E, N-acetylcysteine (NAC), coenzyme Q10, and α-lipoic acid have been extensively investigated in kidney diseases. NAC has shown some benefit in reducing oxidative stress and attenuating contrast-induced nephropathy, although clinical results remain inconsistent [98]. Coenzyme Q10, an essential component of the mitochondrial ETC with antioxidant properties, has demonstrated renoprotective effects in experimental CKD and DKD through reduction in mitochondrial ROS and improvement of mitochondrial respiration. However, despite promising experimental findings, many conventional antioxidants have shown limited efficacy in large clinical trials, potentially due to poor mitochondrial targeting and inadequate modulation of disease-specific signaling pathways [99].

More recently, mitochondria-targeted antioxidants have emerged as a promising therapeutic strategy. Compounds such as MitoQ, SkQ1, and SS-31 (elamipretide) selectively accumulate within mitochondria and directly reduce mtROS production. Experimental studies demonstrated that these agents may preserve mitochondrial membrane integrity, improve ATP production, attenuate inflammation, and reduce fibrosis in AKI and CKD models. SS-31 stabilizes cardiolipin within the inner mitochondrial membrane, thereby improving ETC function and limiting ROS generation [100]. Mitochondria-targeted antioxidants like mitoquinone (MitoQ) and Mito-TEMPO, currently undergoing clinical trials for cardiovascular conditions, lower mtROS levels and inhibit NLRP3 activation in ABS-stimulated human proximal tubule epithelial cells. Inducers of mitophagy, such as urolithin A, spermidine, and rapamycin derivatives, further promote the clearance of mitochondria and mitigate inflammatory signaling. Additionally, Nrf2 activators like bardoxolone methyl also indirectly lessen mtROS-driven NLRP3 activation in ABS-induced rat models [101]. Elamipretide (SS-31), a tetrapeptide that penetrates mitochondria, attaches to cardiolipin to maintain mitochondrial integrity and indirectly reduce ROS production. In the PROGRESS-HF Phase 2 trial, intravenous elamipretide was tolerated well and linked to decreases in left ventricular end-diastolic and end-systolic volumes in patients with heart failure with reduced ejection fraction, even tho al biogenesis and dynamics. Current difficulties include maintaining membrane integrity at elevated doses, ensuring prolonged retention, and comprehending the long-term impacts on mitochondrial dynamics and biogenesis [102].

Restoration of NAD^+^ metabolism represents another important therapeutic approach. Supplementation with NAD^+^ precursors, including nicotinamide riboside (NR) and nicotinamide mononucleotide (NMN), has shown beneficial effects in experimental kidney disease by improving mitochondrial function, enhancing sirtuin activity, and reducing oxidative stress. In AKI models, NAD^+^ repletion improved tubular energy metabolism and accelerated renal recovery, whereas in CKD and DKD it attenuated fibrosis, inflammation, and mitochondrial dysfunction. Inhibition of NAD^+^-consuming enzymes such as PARPs and CD38 has also shown potential in preserving intracellular NAD^+^ levels and improving mitochondrial resilience [103]. NAD^+^ enhancers such as nicotinamide riboside (NR) and nicotinamide mononucleotide (NMN) have transitioned from preclinical to human clinical testing. Animal studies suggest NR and NMN more effectively boost NAD^+^ levels than nicotinic acid (NA) and nicotinamide (NAM), though direct comparisons are lacking. Clinical trials indicate NR can significantly raise NAD^+^ levels in humans, notably a 2.7-fold increase with a 1000 mg dose. A study with 140 participants demonstrated a dose-dependent elevation stabilizing at about twofold after nine days. NR may also improve vascular endothelial function in middle-aged or older adults, with ongoing research into its broader health impacts. NMN is in Phase I trials focusing on safety, insulin sensitivity, endothelial function, and cardiovascular risk indicators, aiming to evaluate its therapeutic potential [104].

Targeting redox-sensitive signaling pathways may additionally provide therapeutic benefit. Activation of the Nrf2 pathway enhances transcription of antioxidant and cytoprotective genes and has demonstrated renoprotective effects in experimental models. Bardoxolone methyl, an Nrf2 activator, initially showed promise in DKD through improvement of eGFR, although concerns regarding cardiovascular safety limited broader clinical application [105]. Modulation of inflammatory and profibrotic signaling pathways downstream of ROS, including NF-κB, TGF-β, and inflammasome activation, also represents a potential therapeutic strategy [106].

Importantly, the dual role of ROS as both signaling molecules and mediators of injury complicates therapeutic intervention. Complete suppression of ROS may disrupt physiological redox signaling and impair adaptive responses. Therefore, future therapies will likely require more selective modulation of pathological oxidative stress while preserving physiological redox signaling mechanisms [101].

### 7.3. Targeting Mitochondrial Function

Because mitochondrial dysfunction represents a central pathogenic mechanism linking AKI, CKD, and DKD, therapeutic approaches aimed at preserving mitochondrial homeostasis and quality control have gained increasing attention. Strategies targeting mitochondrial biogenesis, mitochondrial stress responses, and mitophagy may help restore bioenergetic function and limit progression of kidney injury [107].

Enhancing mitochondrial biogenesis represents one of the most extensively studied approaches. PGC-1α plays a central role in regulating mitochondrial replication, oxidative metabolism, and antioxidant defense. Experimental activation of PGC-1α signaling improved mitochondrial respiration, enhanced fatty acid oxidation, reduced tubular injury, and attenuated fibrosis in AKI and CKD models. Pharmacological agents including AMP-activated protein kinase (AMPK) activators, SIRT1 agonists, and peroxisome proliferator-activated receptor (PPAR) agonists may indirectly stimulate PGC-1α activity and improve mitochondrial function [108].

Targeting the UPR^mt^ has also emerged as a potential therapeutic strategy. Moderate activation of UPR^mt^ may enhance mitochondrial proteostasis, improve antioxidant defense, and increase resistance to stress-induced injury. Experimental induction of UPR^mt^ using nicotinamide riboside, oligomycin, doxycycline, or short-term metabolic stress improved mitochondrial resilience and reduced renal injury in preclinical models [22]. However, the context-dependent effects of UPR^mt^ remain an important challenge, as prolonged or excessive activation may become maladaptive and contribute to persistent inflammation and fibrosis. Consequently, therapeutic modulation of UPR^mt^ will likely require precise temporal and disease-specific control [109]. In recent years, various clinical studies on medications for DKD treatment have shown encouraging outcomes, and many researchers have observed that pharmacological strategies enhance mitochondrial function through the regulation of MQC and the mitigation of DKD. However, despite several therapeutic interventions producing positive effects in DKD management, these treatments still cannot prevent the decline in kidney function,17 highlighting the ongoing urgent need for new prospective strategies for DKD therapy [110,111].

Restoration of mitophagy and mitochondrial turnover also represents a promising approach. Pharmacological enhancement of PINK1-parkin signaling or activation of receptor-mediated mitophagy pathways involving BNIP3 and FUNDC1 has shown beneficial effects in experimental AKI and DKD models by promoting removal of damaged mitochondria, reducing mtROS accumulation, and limiting apoptosis. Agents such as urolithin A, spermidine, metformin, and caloric restriction mimetics may improve mitochondrial quality control and preserve renal mitochondrial integrity [40].

Modulation of mitochondrial dynamics constitutes another emerging therapeutic avenue. Inhibition of excessive DRP1-mediated mitochondrial fission attenuated mitochondrial fragmentation, ROS generation, and tubular apoptosis in experimental AKI. Conversely, restoration of mitochondrial fusion proteins including MFN2 and OPA1 may improve mitochondrial networking and bioenergetic efficiency in CKD states [112].

Despite encouraging preclinical findings, translation into clinical practice remains challenging [110]. Kidney diseases are highly heterogeneous, and mitochondrial responses differ substantially depending on disease stage, metabolic status, and cell type. Furthermore, interventions targeting mitochondrial pathways may produce distinct effects depending on whether mitochondrial stress responses remain adaptive or have already transitioned into maladaptive signaling. The key findings are summarized in Table 3 below.

### 7.4. Translational Considerations

The increasing recognition of mitochondrial–redox dysfunction as a shared pathogenic axis in AKI, CKD, and DKD highlights the need for more individualized therapeutic approaches. Traditional management strategies primarily focus on controlling systemic risk factors and slowing functional decline, whereas emerging therapies aim to target underlying cellular and metabolic mechanisms [20].

Personalized medicine approaches may become particularly relevant because mitochondrial dysfunction and redox imbalance exhibit substantial interindividual variability. Genetic background, age, metabolic status, diabetes, hypertension, and environmental exposures all influence mitochondrial resilience and oxidative stress responses. Consequently, patients may differ considerably in their susceptibility to mitochondrial injury and responsiveness to targeted therapies. Integration of mitochondrial and redox biomarkers with clinical phenotyping may therefore help identify patient subgroups most likely to benefit from specific interventions [113].

In AKI, early identification of patients at risk of maladaptive repair and progression toward CKD remains a major unmet clinical need. Biomarkers reflecting mitochondrial injury, impaired mitophagy, or persistent oxidative stress may facilitate earlier intervention before irreversible fibrosis develops. In CKD and DKD, personalized strategies may involve combining standard renoprotective therapies with agents targeting mitochondrial metabolism, oxidative stress, or mitochondrial quality control pathways [114].

Importantly, currently established therapies may already exert part of their renoprotective effects through modulation of mitochondrial and redox pathways. SGLT2 inhibitors improve mitochondrial efficiency, reduce oxidative stress, and attenuate inflammation in DKD and CKD. Similarly, renin–angiotensin–aldosterone system inhibitors reduce Angiotensin II-mediated ROS production and mitochondrial injury, while mineralocorticoid receptor antagonists may further suppress oxidative and inflammatory signaling [115]. Understanding these mechanisms may facilitate rational combination therapies integrating conventional and mitochondria-targeted approaches.

Several challenges remain before mitochondrial-targeted therapies can be broadly implemented clinically. Many experimental interventions have shown strong efficacy in animal models but limited translation into human studies. The complexity of mitochondrial signaling, disease heterogeneity, and differences between acute and chronic injury states complicate therapeutic development. Moreover, because mitochondrial stress responses may initially serve adaptive functions, inappropriate timing or excessive pathway inhibition could potentially impair protective cellular responses [116]. Table 4 provides an overview of these therapeutic targets and diagnostic biomarkers.

## 8. Conclusions

AKI, CKD, and DKD are no longer viewed as isolated clinical events, but as an interconnected pathological spectrum. At the intersection of these disorders lies a shared mitochondrial–redox axis. Ischemic and metabolic insults trigger an initial wave of cofactor depletion and reductive stress, which rapidly transitions into chronic oxidative stress. This persistent imbalance disrupts cellular signaling, forcing renal cells into abnormal metabolic reprogramming and breaking down the structural integrity of the mitochondrial network.

The ultimate fate of the kidney under these conditions depends on the efficiency of its quality control machinery. While endogenous repair pathways like mitophagy and the UPR^mt^ initially defend the cell, chronic oxidative stress eventually exhausts them. This clearance failure causes damaged mitochondria to accumulate, amplify ROS production, and leak immunogenic fragments into the cytosol—ultimately driving the chronic inflammation and tissue fibrosis that lead to progressive nephron loss.

Translating these insights into clinical practice marks a fundamental paradigm shift from traditional, symptom-driven management toward a mechanism-based approach to personalized nephrology. Standard clinical markers like serum creatinine and albuminuria are retrospectively functional, detecting damage only after substantial tissue decline has occurred, whereas the modern approach focuses on tracking early, upstream cellular stress. Validating non-invasive, multi-marker biomarker panels that monitor mitochondrial injury, co-factor depletion, and macromolecular oxidative damage is essential to capture patients early in the volatile AKI-to-CKD transition.

Concurrently, this mechanistic approach redefines the renal therapeutic landscape. It shifts the focus away from broad, systemic antioxidants toward precise, organelle-targeted interventions designed to preserve cellular energetics. Crucially, this strategy bridges the gap between emerging and established therapies. Rather than replacing the current standard of care, it integrates novel mitochondrial stabilizers and metabolic precursors with breakthrough agents like SGLT2 inhibitors and modern mineralocorticoid receptor antagonists, both of which are now known to achieve their clinical nephroprotection partly by suppressing this exact mitochondrial–redox axis. Combining these therapies to protect the renal energy engine represents the next frontier in halting the global progression of kidney disease.

## Figures and Tables

**Table 1:** Major mitochondrial alterations contributing to bioenergetic dysfunction, oxidative stress, and progression of kidney disease.

Process	Physiological Function	Alteration in Kidney Disease	Consequences
Oxidative phosphorylation (OXPHOS)	ATP production	Impaired ETC activity and ATP depletion [20,21,22,23]	Energetic deficiency and tubular dysfunction
Mitochondrial ROS production	Redox signaling and cellular adaptation	Excess ROS generation [21]	Oxidative stress and mitochondrial damage
Fatty acid β-oxidation	Major energy source for tubular cells	Impaired fatty acid utilization [22,23]	Lipid accumulation and fibrosis
Metabolic reprogramming	Adaptation to metabolic demands	Shift toward glycolysis [24]	Maladaptive repair and chronic injury
UPRmt activation	Maintenance of mitochondrial proteostasis	Insufficient chronic adaptive response [25,26]	Persistent mitochondrial dysfunction
Mitochondrial biogenesis	Renewal of mitochondrial network	Reduced PGC-1α signaling [27,28,29]	Impaired recovery and fibrosis
Mitochondrial dynamics	Maintenance of mitochondrial integrity	Excess fission and reduced fusion [31,32,33,34]	Fragmentation, apoptosis, and tubular injury

Abbreviations: ETC, electron transport chain; ROS, reactive oxygen species; UPRmt, mitochondrial unfolded protein response; PGC-1α, peroxisome proliferator-activated receptor gamma coactivator-1 alpha.

**Table 2:** Acute versus chronic roles of UPRmt in AKI, CKD and DKD.

Feature	AKI	CKD	DKD
Main stress context	Acute mitochondrial injury, ischemia–reperfusion, nephrotoxins, ATP depletion [49]	Chronic mitochondrial stress, inflammation, oxidative injury, and impaired mitochondrial adaptation [53,54,55]	Chronic hyperglycemia, metabolic overload, redox imbalance, and mitochondrial proteotoxic stress [48,56]
Early/adaptive UPRmt role	Transient UPRmt activation may support mitochondrial proteostasis, antioxidant defense, and tubular epithelial cell survival [50,51]	Adequate mitochondrial stress adaptation, including LONP1-related proteostasis, may limit mitochondrial dysfunction [53,54]	Initial activation of ATF5/HSP60/LONP1 related pathways may compensate for hyperglycemia—induced mitochondrial injury [48]
Maladaptive transition	If injury is severe or sustained, UPRmt may become insufficient, contributing to tubular cell death and maladaptive repair [52]	Persistent mitochondrial stress and defective UPRmt-related adaptation may promote mitochondrial dysfunction, inflammation, glomerulosclerosis and fibrosis [53,54,55]	Sustained hyperglycemia may drive prolonged UPRmt activation, mitochondrial ROS accumulation, apoptosis, tubular injury, and tubulointerstitial fibrosis [48,56]
Overall interpretation	Mostly protective when early and transient, potentially harmful when overwhelmed	Mainly reflects insufficient adaptation to chronic mitochondrial injury	Initially compensatory, but potentially maladaptive under chronic diabetic stress

Abbreviations: UPRmt, mitochondrial unfolded protein response; AKI, acute kidney injury; CKD, chronic kidney disease; DKD, diabetic kidney disease. References correspond to studies discussed in Section 5.2.

**Table 3:** Emerging mitochondrial-targeted therapeutic strategies for kidney disease: mechanisms of action, representative clinical trials, and major limitations to clinical translation [100,101,102,103,104,105,106].

Therapeutic Approach	Mechanism of Action	Representative Clinical Trials	Major Limitations to Clinical Use
SS-31 (Elamipretide)	Binds cardiolipin, stabilizes the inner mitochondrial membrane, improves ETC function, and reduces mitochondrial ROS production.	Phase II PROGRESS-HF trial; clinical studies in primary mitochondrial disorders; no established renal trials.	Lack of kidney-specific randomized clinical trials; intravenous administration; long-term efficacy and safety remain uncertain.
MitoQ (mitoquinone)	Mitochondria-targeted antioxidant that scavenges mitochondrial ROS and preserves mitochondrial function.	Early-phase clinical studies in cardiovascular and metabolic diseases; no completed efficacy trials in AKI, CKD, or DKD.	Limited kidney-specific clinical evidence; optimal dosing and long-term efficacy remain unknown.
Nicotinamide riboside (NR)	NAD^+^ precursor that enhances mitochondrial bioenergetics and activates sirtuin signaling.	Phase I/II trials demonstrating increased NAD^+^ levels and favorable safety; no definitive renal outcome trials.	Lack of large randomized clinical trials in kidney disease; clinical efficacy remains uncertain.
Nicotinamide mononucleotide (NMN)	NAD^+^ precursor that improves mitochondrial metabolism and redox homeostasis.	Early Phase I clinical trials evaluating safety, insulin sensitivity, and vascular function.	No efficacy studies in kidney disease; optimal dosage and long-term safety remain unclear.
Bardoxolone methyl (Nrf2 activator)	Activates Nrf2-mediated antioxidant and cytoprotective signaling pathways.	BEAM, BEACON, TSUBAKI, and AYAME trials in CKD and	

Abbreviations: AKI—acute kidney injury; CKD—chronic kidney disease; DKD—diabetic kidney disease; ETC—electron transport chain; NAD^+^—nicotinamide adenine dinucleotide; Nrf2—nuclear factor erythroid 2-related factor 2.

**Table 4:** Translational Strategies and Biomarkers Targeting the Mitochondrial-Redox Axis in Renal Injury.

Focus Area	Targeting	Key Examples	The Main Goal
Early Detection (Biomarkers)	Cellular and DNA damage [90,91] Leaked mitochondrial fragments [92] Energy cofactor depletion [93]	MDA, 8-OHdG [90,91], urinary mtDNA [92], NAD^+^ levels [93]	Detect kidney injury prior to traditional functional markers like serum creatinine [95].
Balancing Redox (Antioxidants and Pathways)	Mitochondrial-targeted scavengers [100] Cytoprotective signaling pathways [105]	SS-31 (elamipretide), MitoQ [96], Nrf2 activators [105]	Neutralize pathological ROS overproduction while preserving physiological redox signaling [101].
Repair and Quality Control (Mitochondrial Function)	Clearance of dysfunctional organelles [20] Mitochondrial biogenesis pathways [108]	Metformin, Urolithin A [20], AMPK activators [108]	Promote the clearance of dysfunctional mitochondria via mitophagy [20] and stimulate mitochondrial biogenesis [108].
Current Standard Care (Established Therapies)	Systemic protection with secondary mitochondrial benefits [115]	SGLT2 inhibitors, RAAS inhibitors [115]	Optimize renal mitochondrial energetics by integrating established standard-of-care therapies [115].

Abbreviations: 8-OHdG, 8-hydroxy-2′-deoxyguanosine; AMPK, AMP-activated protein kinase; MDA, malondialdehyde; mtDNA, mitochondrial DNA; NAD^+^, nicotinamide adenine dinucleotide; Nrf2, nuclear factor erythroid 2-related factor 2; RAAS, renin–angiotensin–aldosterone system; ROS, reactive oxygen species; SGLT2, sodium-glucose cotransporter 2.

## Data Availability

No new data were created or analyzed in this study. Data sharing is not applicable to this article.

## Abbreviations

The following abbreviations are used in this manuscript:

4-HNE	4-Hydroxynonenal
8-OHdG	8-Hydroxy-2′-deoxyguanosine
AGE	Advanced Glycation End-product
AKI	Acute Kidney Injury
AMBRA1	Autophagy Stabilizing Regulator of BECN1
AMPK	AMP-activated Protein Kinase
AOPPs	Advanced Oxidation Protein Products
ATP	Adenosine Triphosphate
BNIP3/BNIP3L	BCL2 Interacting Protein 3/BCL2 Interacting Protein 3 Like
CGA	Cause, Glomerular Filtration Rate, and Albuminuria (System)
CKD	Chronic Kidney Disease
DAMP	Damage-Associated Molecular Pattern
DKD	Diabetic Kidney Disease
DRP1	Dynamin-Related Protein 1
eGFR	Estimated Glomerular Filtration Rate
ETC	Electron Transport Chain
FADH_2_	Flavin Adenine Dinucleotide (reduced form)
FUNDC1	FUN14 Domain Containing 1
HUWE1	HECT, UBA, and WWE Domain Containing E3 Ubiquitin Protein Ligase 1
IRI	Ischemia–Reperfusion Injury
KDIGO	Kidney Disease: Improving Global Outcome
LC3/LC3B/LC3II	Microtubule-associated Protein 1 Light Chain 3 (B/II)
MAPK	Mitogen-Activated Protein Kinase
MDA	Malondialdehyde
MFN1/MFN2	Mitofusin 1/Mitofusin 2
mtDNA	Mitochondrial DNA
mtROS	Mitochondrial Reactive Oxygen Species
MUL1	Mitochondrial E3 Ubiquitin Protein Ligase 1
NAC	N-Acetylcysteine
NAD^+^/NADH	Nicotinamide Adenine Dinucleotide (oxidized/reduced form)
NADPH	Nicotinamide Adenine Dinucleotide Phosphate
NLRP3	NLR Family Pyrin Domain Containing 3
NMN	Nicotinamide Mononucleotide
NOX	NADPH Oxidase
NR	Nicotinamide Riboside
Nrf2	Nuclear Factor Erythroid 2-Related Factor 2
NSAIDs	Nonsteroidal anti-inflammatory drugs
OPA1	Optic Atrophy Protein 1
OXPHOS	Oxidative Phosphorylation
PARPs	Poly(ADP-ribose) Polymerases
PGC-1α	Peroxisome Proliferator-Activated Receptor Gamma Coactivator-1 Alpha
PINK1	PTEN-induced Kinase 1
PPAR	Peroxisome Proliferator-Activated Receptor
PRKN	Parkin RBR E3 Ubiquitin Protein Ligase
PTMs	Post-Translational Modifications
RAAS	Renin–Angiotensin–Aldosterone System
ROS	Reactive Oxygen Species
SGLT2	Sodium-Glucose Cotransporter 2
TCA	Tricarboxylic Acid Cycle
TFAM	Mitochondrial Transcription Factor A
UPR^mt^	Mitochondrial Unfolded Protein Response
